# Programmed Death–Ligand 1 and Vimentin: A Tandem Marker as Prognostic Factor in NSCLC

**DOI:** 10.3390/cancers11101411

**Published:** 2019-09-22

**Authors:** Julien Ancel, Philippe Birembaut, Maxime Dewolf, Anne Durlach, Béatrice Nawrocki-Raby, Véronique Dalstein, Gonzague Delepine, Silvia Blacher, Gaëtan Deslée, Christine Gilles, Myriam Polette

**Affiliations:** 1 Inserm, Université de Reims Champagne Ardenne, P3Cell UMR-S1250, SFR CAP-SANTE, 51097 Reims, France; 2 Service de pneumologie, Hôpital Maison Blanche, CHU de Reims, 51092 Reims, France; 3 Laboratoire de biopathologie, Hôpital Maison Blanche, CHU de Reims, 51092 Reims, France; 4 Service de chirurgie cardio-vasculaire et thoracique, Hôpital Robert Debré, CHU de Reims, 51092 Reims, France; 5 Laboratory of Tumor and Development Biology, GIGA-Cancer, University of Liège, 4000 Liège, Belgium

**Keywords:** epithelial–mesenchymal transition, Programmed Death–Ligand 1, Non-Small-Cell Lung Cancer, vimentin, immune checkpoints

## Abstract

In non-metastatic non-small-cell lung cancer (NSCLC), outcomes remain poor. Adjuvant chemotherapies provide a limited improvement in disease-free survival. Recent exploratory studies on early-stage NSCLC show that immunotherapy given according to Programmed Death–Ligand 1 expression generates variable results, emphasizing a need to improve tumor characterization. We aimed to conjointly assess NSCLC, the expression of PD–L1, and epithelial–mesenchymal transition, frequently involved in tumor aggressiveness. 188 resected NSCLCs were analyzed. Among 188 patients with curatively resected NSCLC, 127 adenocarcinomas and 61 squamous cell carcinomas were stained for PD–L1 and vimentin expression. Overall survival has been compared regarding PD–L1 and vimentin statuses both separately and conjointly in Tumor Cancer Genome Atlas databases. PD–L1 and vimentin higher expressions were strongly associated (*OR* = 4.682, *p* < 0.0001). This co-expression occurred preferentially in tumors with lymph node invasion (*p* = 0.033). PD–L1 was significantly associated with high EMT features. NSCLC harboring both PD–L1^high^/vimentin^high^ expressions were significantly associated with poor overall survival (*p* = 0.019). A higher co-expression of vimentin and PD–L1 was able to identify patients with worse outcomes. Similar to an important prognostic marker in NSCLC, this tandem marker needs to be further presented to anti-PD–L1 immunotherapies to improve outcome.

## 1. Introduction

Despite therapeutic advances, lung cancer remains the leading cause of death by cancer. Over the past few years, immunotherapy has become a major treatment in the management of non-small-cell lung cancer (NSCLC). PD–1/PD–L1 (Programmed Death–Ligand 1) is an important inhibitory checkpoint used by both tumor cells and/or tumor-infiltrating immune cells hindering anti-tumor immune response. PD–L1 expression is a marker used in daily clinical practice to guide immunotherapy management. The fast progression of immunotherapies has led to an improvement in the management of metastatic NSCLC. As published in KEYNOTE-024 for Pembrolizumab [1], CHECKMATE-017 for Nivolumab [2,3], progression-free survival (PFS) and overall survival (OS) are significantly improved by these treatments. However, those treatments are not recommended in non-metastatic conditions where surgical resection remains the gold-standard management. Nevertheless, using this surgical approach, 5-year survival rates drops from 60% for stage IIA disease to 36% for stage IIIA disease, according to the 8th edition staging project of the International Association for the Study of Lung Cancer (IASLC) [4]. Taking into consideration poor outcomes, even after a complete surgical resection, adjuvant strategies have been designed. In daily practice, adjuvant platinum-based chemotherapy shows a limited improvement of 5% in survival at 5 years for completely resected stage II–III NSCLC [5]. Aiming at improving outcomes, immune checkpoint inhibitors have been tested, though limited data are available on immunotherapy in early-stage NSCLC. Recently, Forde et al demonstrated the safety and feasibility of using Nivolumab in this context with only a few partial responses [6]. Elsewhere, the PACIFIC study showed an improvement in PFS in a narrowly selected cohort of patients who received chemoradiotherapy pretreatment. Even in doing so, the response rate only reached 28.4% [7]. Others phase III trials in early-stage NSCLC with immunotherapies are still in progress (NCT02595944, NCT02504372). Taken together, those trials highlight feasibility and potential activity of anti-PD–L1 blockade in early-stage NSCLC. However, response rates remain lower than expected and emphasize the need to refine tumor characterization, to better predict disease outcomes in non-metastatic condition and improve patient stratification for clinical management. Epithelial–mesenchymal transition (EMT), described as a marker of aggressiveness in NSCLC, appears to be a good candidate to help refinement of tumor characterization.

EMT is characterized by diminished epithelial characteristics and enhanced mesenchymal attributes. This process provides tumor cells with enhanced invasive and survival properties and have been associated with tumor invasion, circulating tumor cells, and metastatic colonization [8]. Thus, EMT features (such as increased expression of vimentin or EMT core transcription factors and cell–cell contact remodeling) have been associated with poor clinical parameters in a variety of cancers including NSCLC [9]. Vimentin, a canonical marker and actor of EMT, has accordingly been extensively described as a prognostic marker in various cancers [10,11]. In NSCLC, our laboratory [12] demonstrated that vimentin can predict the occurrence of metastases. New therapeutic strategies targeting EMT are thus being developed in clinical studies but have not yet been approved for use in clinical practice [13,14].

Considering PD–L1 as a marker associated with immunotherapy response and immune escape in NSCLC and vimentin as marker of aggressiveness, we proposed that both PD–L1 and vimentin could be analyzed to refine tumor characterization and stratification at the early stage. Indeed, a better tumor characterization using established prognostic factors along with known drug targets may help identify patients with suspected worse outcomes at early stages and further refine patient selection for anti-PD–L1 treatments. With this in mind, we aimed to examine the potential correlation between vimentin and PD–L1 expression in NSCLC and their respective impacts on patient outcomes.

## 2. Results

### 2.1. Characteristics Baselines

Both males and smokers were more represented. Adenocarcinoma (AC) (*n* = 127) was the major histological type represented, followed by squamous cell carcinoma (SCC) (*n* = 61). Characteristics of tumor sizes (pT) and lymph node status (pN) are available in Appendix Table A1.

### 2.2. Programmed Death–Ligand 1 Expression

**Figure 1:**
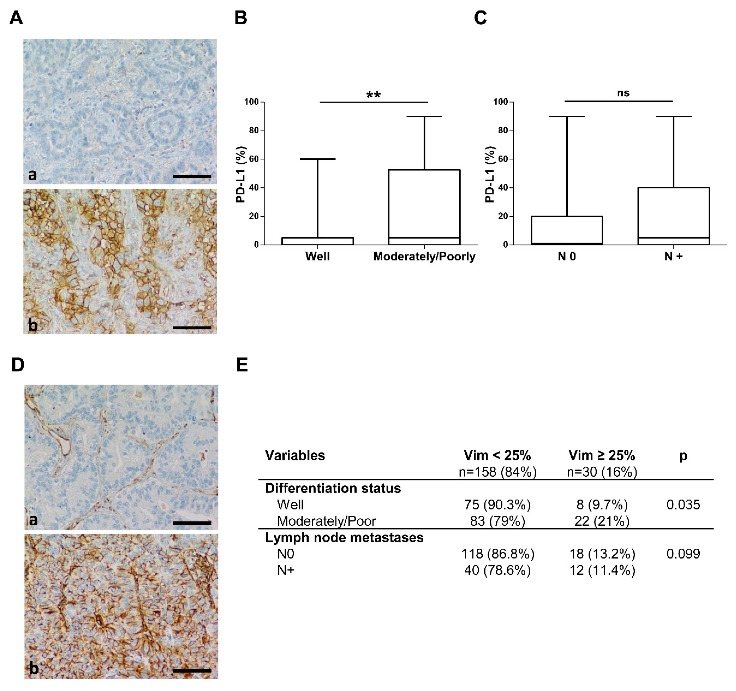
PD–L1 and vimentin expressions in NSCLC. (**A**) Illustration of PD–L1 immunostaining performed on well-differentiated (**a**) and on poor-differentiated adenocarcinomas (**b**). (Bar = 60 µm). (**B**) PD–L1 expression by tumor differentiation degree. Tumors samples divided into well (*n* = 83) and moderately/poorly (*n* = 105) differentiated. (**C**) PD–L1 expression by lymph node status. Tumors samples distributed by lymph node invasion. (**D**) Illustration of vimentin immunostaining performed on well-differentiated (**a**) and on poor-differentiated adenocarcinomas (**b**) (Bar = 60 µm). (**E**) Vimentin expression by tumor differentiation degree and lymph node status. Data in the boxplots are presented as median ± 95 percentile and table of contingency. ** *p* < 0.01.

**Figure A1:**
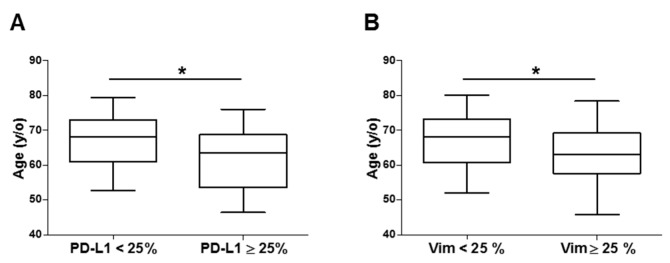
PD–L1 was correlated with age. (**A**) Younger cases expressed higher PD–L1 level. Tumors samples divided into high (*n* = 47) and low (*n* = 141) PD–L1 expression. (**B**) Younger cases expressed higher vimentin level. Tumors samples divided into high (*n* = 30) and low (*n* = 158) vimentin expression. Data are presented with boxplots as median ± 95 percentile. * *p* < 0.05.

101 tumors (54%) were PD–L1-positive including 47 tumors (25%) with ≥25% PD–L1-positive tumor cells and a mean of 18.4 ± 30%. PD–L1 expression was associated with tumor cell dedifferentiation (*p* = 0.009). Indeed, moderately and poorly differentiated tumors more frequently expressed PD–L1 over 25% (*OR* = 2.879). Poorly and moderately differentiated tumors had a higher PD–L1 expression than well-differentiated tumors (*p* = 0.002) with PD–L1 levels reaching 25.2% and 9.7%, respectively (Figure 1A,B). With a PD–L1 threshold at 25%, patients with a higher PD–L1 expression were younger (Appendix Figure A1A) (61.8 ± 8.8 vs. 66.8 ± 8.2 years respectively, *p* = 0.016). No association between PD–L1 and gender, tumor size, stage disease, histological type, or especially with lymph node invasion were found (Figure 1C).

### 2.3. Vimentin Expression

Ninety tumors (48%) were vimentin-positive including 30 tumors (11%) with ≥25% vimentin-positive tumor cells. Vimentin expression was associated with tumor cell dedifferentiation (*p* = 0.035). Indeed, moderately and poorly differentiated tumors more frequently expressed vimentin over 25% (*OR* = 2.485) (Figure 1D,E). A higher level of vimentin was more frequently observed in younger patients (*p* = 0.047) (Appendix Figure A1B). There was no significant association between vimentin expression and gender, tumor size, histological type, lymph node metastases, or clinical-stage disease (Figure 1E).

### 2.4. PD–L1 and Vimentin Co-Expression

**Figure 2:**
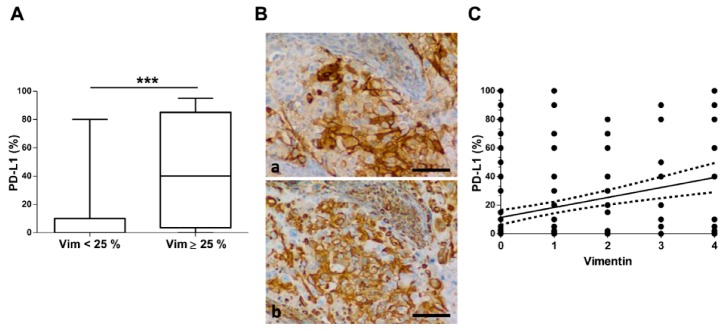
PD–L1 and vimentin association in NSCLC. (**A**) PD–L1 repartition by vimentin expression. High vimentin expression was considered with ≥25% (*n* = 30) tumor cell immunostained and low for others (*n* = 158). Data in the boxplots are presented as median ± 95 percentile. *** *p* < 0.001. (**B**) PD–L1 and vimentin expression are co-expressed in tumor cells. Histopathological illustration of PD–L1 and vimentin expression on adenocarcinoma (Bar = 60 µm). (**C**) Vimentin expression is correlated with PD–L1 expression in NSCLC. (*r* = 0.300; *p* < 0.0001). Regression line with confidence interval _95_CI (dashed curves) are represented.

There was a significant association between PD–L1 and vimentin positivity. The level of PD–L1 expression was three times more frequent in the positive vimentin group (*p* = 0.011). In tumors with ≥25% vimentin-positive tumor cells, numerous tumor cells co-expressed vimentin and PD–L1. Moreover, there was a higher PD–L1 level of expression when ≥25% tumor cells were positive for vimentin (14 ± 25 vs. 43 ± 38 PD–L1 positive cells; *p* < 0.0001) (Figure 2A,B).

Examining the co-expression of PD–L1 and vimentin, a positive correlation was observed in the univariate PD–L1 linear regression by vimentin (*r* = 0.300; *p* < 0.0001) (Figure 2C). With a threshold of 25% positive cell for both PD–L1 and vimentin, this association was enhanced. Indeed only 19% (*n* = 31) tumors with a low vimentin expression had more than 25% PD–L1 positive cells while 53% (*n* = 16) tumors with ≥25% vimentin-positive cells expressed ≥25% PD–L1 positive cells (*OR* = 4.682, *p* < 0.0001). PD–L1 and vimentin co-expression was observed in same proportion for both AC and SCC (*p* = 0.075).

In univariate analysis, co-expression of PD–L1 and vimentin were influenced by age, tumor grade differentiation, and lymph node metastasis. Finally, in multivariate analysis, poor differentiation (*p* = 0.011) and lymph node invasion (*p* = 0.033) were significantly associated with PD–L1 and vimentin co-expression whatever the histological types (*p* = 0.100). Indeed, in an advanced context as illustrated by lymph node invasion, tumors more frequently and strongly co-expressed both PD–L1 and vimentin (*OR* = 3.549, *p* = 0.033) (Table 1).

### 2.5. Correlation of PD–L1 with EMT in Tumor Cancer Genome Atlas (TCGA) and Impact on Overall Survival

**Figure 3:**
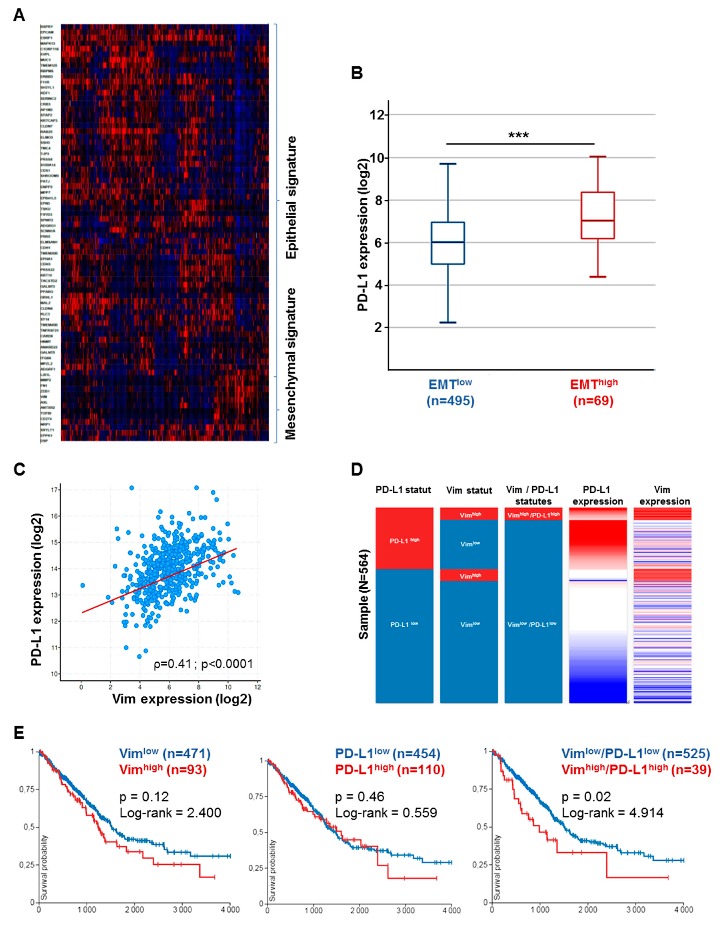
The epithelial–mesenchymal transition signature correlates with PD–L1 expression in Tumor Cancer Genome Atlas Lung Adenocarcinoma (TCGA–LUAD) cohort. (**A**) Heat map showing the differential gene expression by epithelial–mesenchymal features in TCGA–LUAD cohort. (**B**) PD–L1 expression is higher in mesenchymal-types tumors. Tumors with epithelial phenotype (EMT^low^) (*n* = 495) expressed lower mRNA CD274 level than tumors with mesenchymal features (EMT^high^) (*n* = 63). *** *p* < 0.001. (**C**) PD–L1 correlates with vimentin expression in TCGA cohort. Data are showed with dot-plots with log2 scale and analyzed with spearman regression test. (**D**) Tumor repartition in LUAD cohort according to PD–L1 and/or vimentin status. (**E**) Survival analysis according to vimentin, PD–L1 expressions and both Vim/PD–L1 co-expression. Overall survival probability by time (days) represented with Kaplan–Meier curves.

First, we presented PD–L1 expression with a 75-gene EMT expression signature obtained from the GSE4824 dataset, which predicted mesenchymal features in lung cancer cell lines. Indeed, a heat map managed to identify common overexpressed genes in mesenchymal conditions such as ZEB1, AXL, FN1, MMP2, CDH3, and VIM. Then, tumors with an epithelial signature expressing TJP, MUC1, and CDH1 that encodes E-cadherin were discriminated from mesenchymal ones. Regarding CD274 gene expression that encodes PD–L1, it was found in the same cluster as other genes highly expressed in tumors of mesenchymal features (Figure 3A). Interestingly, PD–L1 expression was more significantly higher in tumors with mesenchymal features than those with epithelial features in AC cohort (*p* < 0.0001; Figure 3B). Out of 564 cases, 110 (19.5%) tumors were considered to be PD–L1^high^ while 93 tumors (16.5 %) were considered with a high mesenchymal pattern as observed in our cohort. Concerning co-expression genes by linear regression, PD–L1 expression positively correlated with many genes highly expressed during mesenchymal transition such as VIM, which encodes vimentin (*r* = 0.5; *p* < 0.0001) (Figure 3C), FN1, which encodes fibronectin (*r* = 0.4; *p* < 0.0001), and ZEB1, which encodes Zinc finger E-box-binding homeobox 1 (*r* = 0.34; *p* < 0.0001).

**Figure 4:**
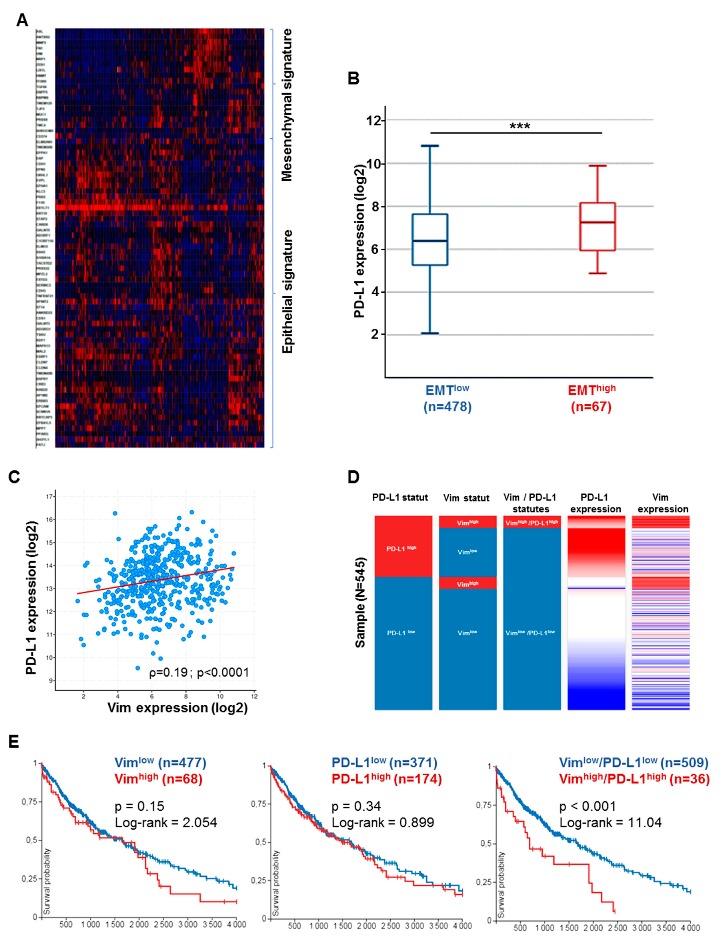
The epithelial–mesenchymal transition signature correlates with PD–L1 expression in Tumor Cancer Genome Atlas Lung Squamous Cell Carcinoma (TCGA–LUSC) cohort. (**A**) Heat map showing the differential gene expression by epithelial–mesenchymal features in TCGA–LUSC cohort. (**B**) PD–L1 expression is higher in mesenchymal-types tumors. Tumors with epithelial phenotype (EMT^low^) (*n* = 478) expressed lower mRNA CD274 level than tumors with mesenchymal features (EMT^high^) (*n* = 67). *** *p* < 0.0001. (**C**) PD–L1 correlates with vimentin expression in TCGA cohort. Data are showed with dot-plots with log2 scale and analyzed with spearman regression test. (**D**) Tumors’ repartition in LUSC cohort according to PD–L1 and/or vimentin status. (**E**) Survival analysis according to vimentin, PD–L1 expressions and both Vim/PD–L1 co-expression. Overall survival probability by time (days) represented with Kaplan–Meier curves.

Considering prognostic value, high vimentin phenotype was not able to distinguish different outcomes (OS50 = 1500 vs. 1150 days, *p* = 0.12; Log-rank = 2.4). Likewise, the same observation was made for PD–L1 expression in adenocarcinoma cohort (*p* = 0.46). On the contrary, taking into account both high PD–L1 and high EMT co-expression was associated with different patient outcomes. Vim ^high^/PD–L1^high^ tumors had a significantly worse evolution in OS than Vim^low^/PD–L1^low^ tumors (Log-rank = 4.9, *p* = 0.02) with median OS respectively at 1000 and 1650 days (Figure 3D,E). The same observations were made for SCC (TCGA–LUSC), for both PD–L1 co-expression and patient outcomes (Figure 4A–E).

## 3. Discussion

We examined PD–L1 and vimentin expression in a population of 188 patients eligible for surgical treatment. Our study thus clearly demonstrates a strong significant association in NSCLC between PD–L1 and vimentin that correlates better with worse clinical parameters than each marker considered individually. Our results are supported by a recent study reported by Kaifi et al. [15]. Using circulating tumor cells (CTCs) in 30 resected NSCLCs, this study shows PD–L1 expression and EMT of CTCs is a negative survival predictor for NSCLC patients. However, the CTC approach does not take into account tumor heterogeneity. Our study, conducted on whole tissue sections, allows consideration of this parameter, improving correlative analyses of heterogeneous biomarkers such as PD–L1 and vimentin and also represents the largest cohort on the subject.

Considering PD–L1 separately, our study shows that its expression was negatively correlated with age, contrary to previous studies [16,17]. The fact that those studies focused on metastatic NSCLC may explain the difference with our observations. A correlation between PD–L1 expression and age has also been described in other extra-thoracic histological types [18]. Our analyses further showed no association between PD–L1 and gender, smoking history, primitive tumor site, which is consistent with the literature [19]. There was no difference of PD–L1 expression in adenocarcinomas and squamous cell carcinomas as also previously reported [20,21]. PD–L1 was associated with a poorly differentiated phenotype in all tumors. As reported by Lin et al, our observations confirm that PD–L1 expression by itself therefore appears not sufficient as a prognostic factor [22]. This is in contradiction to the study by Kim et al, reporting that PD–L1 alone may be a poor prognostic factor in early-stage lung carcinomas, with a cut-off value of 1% [23]. Regarding vimentin, previous works have pointed out the interest of its detection in tumor cells as a relevant marker of EMT and tumor aggression [24,25]. Our present study confirms these data with a threshold ≥25% of positive tumor cells significantly associated with poor tumor differentiation. Moreover, and similarly to PD–L1, vimentin alone appears insufficient to predict patient outcomes.

Importantly, our data show a strong association between vimentin and PD–L1 as previously reported in human esophageal [26] and breast cancers [27,28]. In most of the vimentin-positive carcinomas, numerous tumor cells co-expressed vimentin and PD–L1, supporting a narrow relationship between EMT and PD–L1 confirmed in both proper and TCGA cohorts. Moreover, a clinically relevant interrelation between vimentin and PD–L1 expression has been demonstrated, notably in head and neck squamous carcinoma [29]. Accordingly, in a recent paper, Asgarova et al. [30] demonstrated that EMT may control the expression of PD–L1, promoting immune evasion. They also observed an overexpression of PD–L1 in vimentin-positive NSCLC tissues in a cohort of 40 patients. This clinical observation is in favor of a relation between PD–L1 and EMT, as shown in the pre-clinical model. Using a model of reversible EMT based on a TNFα/TGFβ treatment of the lung cancer cell line A549, they showed an up-regulation of PD–L1, with a demethylation of its promoter via the NFκB pathway [30]. In another study, Noman et al. showed an up-regulation of PD–L1 in an EMT-activated human breast cancer cell by a mechanism involving ZEB1 and MIR-200 [31]. Using murine models of lung carcinoma, Li et al. proposed the intervention of ERK, ALK, and TAK pathways to explain the regulation of PD–L1 expression during the EMT processes [32]. Alternatively, modulating PD–L1 expression was also shown to regulate EMT in human esophageal cancer cells [26]. Interestingly, Tripathi et al. described in NSCLC a low expression of immunoproteasome in NSCLC tumors with mesenchymal features. Anti-PD–L1 molecules could thus help to overcome immune evasion [33]. All these data emphasize a close relationship between EMT and PD–L1 expression implicating different regulatory mechanisms. Most interestingly, when PD–L1 and vimentin were associated, the correlation with worse prognostic factors (dedifferentiation and lymph node metastases) was stronger. Finally, taking into account both EMT and PD–L1 statuses, high co-expression phenotype appeared as an important prognostic indicator able to identify worse outcomes. One of the main limitations of our study is the retrospective and monocentric design that limits its generalization. Despite the use of independent cohorts for external validation, non-randomized group analysis is exposed to confounding. Moreover, the short-term follow-up in our cohort does not allow the conduction of survival analysis. To confirm our results, prospective randomized trials with adequate design are needed.

Finally, examining PD–L1 expression in association with vimentin as known prognostic marker of aggression in early-stage NSCLC may thus help sub-stratify patients and identify those who would best benefit from adjuvant treatment.

## 4. Materials and Methods

### 4.1. Patient Recruitment and Tumor Staging

NSCLC tumor samples were obtained from 188 patients with primary lung carcinomas who underwent a surgical resection between March 2016 and July 2019 at the University Hospital of Reims. Clinical data such as age, gender, and smoking history were collected retrospectively. This study was conducted in accordance with the ethical guideline of Declaration of Helsinki (NO.DC-2008-374). Surgically resected tumors were collected after obtaining informed consent from patients. Access to patient data for this retrospective non-interventional study was approved by the French national commission for personal data protection (CNIL, Comité National de l’Information et des Libertés) (NO.2049775 v 0). Formalin-fixed and paraffin-embedded tumor samples were obtained from the Tumor Bank of the Reims University Hospital Biological Resource Collection (NO.DC-2008-374) declared at the Ministry of Health according to the French Law, for use of tissue samples for research. The tumors were staged according to the 8th TNM UICC/AJCC edition.

### 4.2. Immunohistochemical Staining

Sections of 4-micrometer were obtained from formalin-fixed paraffin-embedded blocks of NSCLC. Immunohistochemistry was performed on serial tissue sections with antibodies against cytokeratins (AE1/AE3) (Dako Glostrup, Denmark, Ref H3515, dilution 1:50), vimentin (Dako, Ref MO725, dilution 1:600 clone V9), PD–L1 (Dako Glostrup, Denmark, Ref M6353, Clone 22C3, dilution 1:50) using a Ventana Benchmark XT autostainer (Ventana Medical Systems, Inc., Tucson, AZ, USA). Subsequent steps were performed with the UltraView universal DAB detection kit (Ventana). The detection of cytokeratin 7 (Dako, Ref M7018, dilution 1:200) and Thyroid Transcription Factor 1 (TTF1, Neomarker, Fremont, CA, USA, Ref MS699, dilution 1:50) was performed to confirm the diagnosis of primitive adenocarcinoma (AC). Appendix tissue was used as a positive control for vimentin and cytokeratin expression, and tonsil for PD–L1. Negative controls were performed by omitting the primary antibody or incubating with a corresponding IgG isotype. For the detection of PD–L1, we used an antibody (clone 22C3) recommended by the FDA for selecting patients who will receive PD–L1/PD–L1 immunotherapy [16,34]. A blind evaluation of the labeling and a scoring was performed by two independent pathologists. For both vimentin and PD–L1 staining, the percentage of positive tumor cells was scored for each tumor. Vimentin score was established as follows: 0 = no detection, 1 = detection in ≤9% of tumor cells, 2 = detection in 10–24% of tumor cells, 3 = detection in 25–49% of tumor cells, 4 = detection in ≥50% of tumor cells. A threshold of 25% positive tumor cells was considered for the analysis according to the previously reported relevance of this threshold in non-metastatic NSCLC [7].

### 4.3. Selection of Tumor Cancer Genome Atlas (TCGA) Data and EMT Gene Signature

To assess EMT characteristics of tumors from a NSCLC cohort, we used 75 gene expression signatures associated with EMT from a previous study (GSE4824), derived from a NSCLC cell line [35]. Then we downloaded mRNA samples related to lung adenocarcinoma from the Tumor Cancer Genome Atlas database (TCGA–LUAD; *n* = 564) [36] and lung squamous cell carcinoma (TCGA–LUSC; *n* = 545) both composed of a majority of non-metastatic cases. Mean follow-up in LUAD and LUSC cohorts were respectively 29.3 and 31.9 months. Characteristic cohorts are open access and available at: https://portal.gdc.cancer.gov. This gene signature was then applied on a comparison of continuous values, such as the log2 value of reads per kilobase of transcript per million mapped reads (RPKM) of PD–L1, vimentin, and analyzed using a Wilcoxon rank sum test. OS was measured from the diagnosis date until death, or the last follow-up date if censored. Heat maps were generated using cBioPortal [37] to validate EMT signature and PD–L1 (CD274) co-expression. The correlation between EMT/PD–L1 expressions and OS was examined using the University of California Santa-Cruz (UCSC) Xena platform [38].

### 4.4. Statistical Analysis

Data in tables are presented as mean ± standard deviation (SEM). Boxplots represent the median and the quartiles. Association between vimentin +/− and PD–L1 +/− positivity and other features were studied using chi-square. As the Kolmogorov test showed that distributions are not normal, quantitative data were analyzed using a non-parametric Mann–Whitney test to assess significance between different conditions (*p* < 0.05 was considered significant). A Spearman test was used to study the correlation between vimentin and PD–L1. All parameters with a *p*-value < 0.10 in univariate analysis were included in multivariate process. Survival analysis was performed using Kaplan–Meier methods. In all exploratory analyses, results with two-sided *p*-value ≤ 0.05 were considered significant. XLSTAT software (version 2019.1.3, Addinsoft company, Paris, France) was used to analyze and reformat data.

## 5. Conclusions

In conclusion, our study emphasizes a strong correlation between vimentin and PD–L1 expression in NSCLC, particularly in an advanced context. High co-expression phenotype allows identification of worse overall survival. Our approach to co-examine the immune checkpoint marker with the canonical marker of EMT aims to develop a relevant immunoscore in the non-metastatic condition [39,40]. The co-expression of vimentin with PD–L1 may thus represent a promising tandem marker candidate to further refine patient selection and precise eligibility for adjuvant treatment, especially in the immune checkpoint area.

## Figures and Tables

**Table 1:** Factors associated with PD–L1 and vimentin co-expression in NSCLC.

Variables	Univariate Analysis			Multivariate Analysis	
	OR	OR–CI 95	*p*-Value	OR	*p*-Value
**Age**	0.940	0.885–0.997	0.038		0.308
**Histological type**					
Adenocarcinoma	1				
Squamous cell carcinoma	0.274	0.060–1.244	0.094		0.100
**Tumor grade differentiation**					
Well	1		-		
Moderately	2.761	0.445–17.153	0.276		0.321
Poorly	9.479	2.014–44.610	0.004	8.181	0.011
**Primitive tumor size, pT**					
T1	1		-		
T2	1.058	0.318–3.513	0.927		
T3	1.000	0.194–5.162	1.000		
T4	1.048	0.202–5.422	0.956		
**Lymph node status, pN**					
N0	1		-		
N+	3.013	1.031–8.802	0.044	3.549	0.033

PD–L1: Programmed Death–Ligand 1; OR: Odd Ratio.

## Appendix A

**Table A1:** Characteristics of the study population.

Variables	Patients (*n* = 188)
**Sex (male/female)**	124/64
**Age (y)**	66 ± 8
**Smoking history**	
Non-smokers	8% (15)
Former smokers	31% (58)
Current smokers	48% (90)
Unknown	13% (24)
Pack-years	37 ± 23
**Histological type**	
Adenocarcinoma	68% (127)
Squamous cell carcinoma	32% (61)
**Tumor Grade differentiation**	
Poorly	31% (58)
Moderately	25% (47)
Well	44% (83)
**Tumor size**, pT	
T1	45% (84)
T2	30% (57)
T3	13% (24)
T4	12% (23)
**Lymph node status**, pN	
N0	66% (123)
N1	11% (20)
N2	17% (32)
Nx	6% (12)

Values are mean ± standard deviation or percentage (number).
